# Physical fitness factors associated with change-of-direction performance in primary schoolchildren: the role of muscular strength, flexibility, and sex

**DOI:** 10.3389/fspor.2026.1972450

**Published:** 2026-10-14

**Authors:** María del Carmen Carcelén-Fraile, Agustín Aibar-Almazán, Laura Peris-Reig, Noelia Vigil-Torres

**Affiliations:** 1 Department of Educational Sciences, Faculty of Social Sciences, University of Atlántico Medio, Las Palmas de Gran Canaria, Spain; 2 Department of Health Sciences, Faculty of Health Sciences, University of Jaén, Jaén, Spain

**Keywords:** change of direction, explosive strength, flexibility, muscular strength, physical fitness, schoolchildren

## Abstract

**Introduction:**

Change-of-direction (COD) performance is an important component of motor performance during childhood and depends on multiple physical capacities. However, the associations of muscular strength, muscular endurance, and flexibility with COD performance, and the potential moderating role of sex, remain insufficiently understood in school-aged children.

**Methods:**

This cross-sectional study included 386 primary schoolchildren (176 boys and 210 girls; mean age: 11.15 ± 0.66 years). Handgrip strength, lower-body explosive strength, trunk muscular endurance, flexibility, and COD performance were assessed using handgrip dynamometry, the standing long jump, 30-s sit-up, sit-and-reach, and 4 x 10-m shuttle-run tests, respectively. Sex differences were examined using independent-samples t-tests. Pearson correlations and hierarchical multiple linear regression analyzes examined associations with COD performance, with interaction terms used to assess moderation by sex. Not statistically significant Differences between boys and girls were detected in physical fitness components or COD performance (all *p* > 0.05).

**Results:**

COD performance was significantly associated with lower-body explosive strength (r = −0.775), handgrip strength (r = −0.706), flexibility (r = −0.534), and trunk muscular endurance (r = −0.532) (all *p* < 0.001). After adjustment for age, sex, and BMI, physical fitness components increased the explained variance in COD performance by 61.4% (*Δ*R^2^ = 0.614, *p* < 0.001), with the final model explaining 62.6% of the variance. Lower-body explosive strength showed the strongest independent association (*β* = −0.566, *p* < 0.001), followed by handgrip strength (*β* = −0.180, *p* = 0.003) and flexibility (*β* = −0.111, *p* = 0.005). Trunk muscular endurance was not independently associated with COD performance (*β* = 0.007, *p* = 0.878). Sex did not mean anything moderated these associations (*Δ*R^2^ = 0.003, *p* = 0.632).

**Conclusion:**

Physical fitness, particularly lower-body explosive strength, was strongly associated with COD performance in primary schoolchildren. Handgrip strength and flexibility showed smaller independent associations, considering trunk muscular endurance did not after simultaneous adjustment for other fitness components. No significant between sex differences or moderating effects of sex were detected.

## 1 Introduction

The transition from childhood to adolescence is a particularly sensitive period for the development of physical fitness and motor skills (1). Coinciding with the final years of primary education, schoolchildren undergo significant transformations associated with growth and biological maturation that affect body composition, musculoskeletal characteristics, neuromuscular control, and the ability to produce force and power (2). Importantly, biological maturation does not occur uniformly, and children of the same chronological age may differ considerably in their maturational status (3). Furthermore, their expression differs progressively between boys and girls, especially as pubertal maturation progresses. Recent evidence indicates that strength and power increase during childhood and tend to accelerate around puberty, particularly in boys, as a result of changes in muscle mass, tendon stiffness, fat-free mass, and neuromuscular function (4). Recent population studies also show that sex differences in various manifestations of physical fitness widen during adolescence, reflecting divergent maturational trajectories (5).

Within this developmental process, motor performance emerges from the interaction of multiple individual, task, and environmental constraints (6). Pre-planned change-of-direction (COD) tasks represent a complex motor demand, requiring rapid deceleration, control of body position, and subsequent force production to reaccelerate the body in a new direction (7). Accordingly, COD performance is associated with multiple biomechanical and neuromuscular characteristics rather than with a single physical capacity (7). Evidence in young populations further indicates that multidirectional performance develops throughout childhood and adolescence and may vary according to age and maturational status (8). Identifying the physical fitness characteristics associated with COD performance is therefore relevant for understanding motor performance during school age. Among these physical fitness components, muscular strength may be particularly relevant to COD performance. Rapid changes of direction require substantial force production during braking and subsequent reacceleration, placing considerable demands on the lower limbs (7, 9). Evidence from primary schoolchildren also indicates that lower-limb power, sprint speed, and multidirectional motor performance are closely related components of physical fitness that develop throughout the primary-school years (10). Evidence from training studies further supports the relationship between lower-body strength and power and COD performance; however, much of this research has been conducted in trained adolescent athletes. For example, a systematic review and meta-analysis of highly trained male youth soccer players found that strength, plyometric, and combined training improved lower-body strength, horizontal power, acceleration, and COD speed (11). Therefore, although the available evidence supports a close relationship between muscular strength and COD performance, the extent to which findings derived from trained adolescent athletes apply to younger children from general school populations remains unclear. However, focusing exclusively on lower-body strength may provide an incomplete characterization of muscular fitness. Different manifestations of muscular strength reflect partly distinct aspects of neuromuscular development and may exhibit different developmental patterns across childhood and adolescence (12). Evidence from children and adolescents indicates that sex differences in muscular strength are already present during childhood but become substantially larger during adolescence, with the magnitude of these differences also varying between upper- and lower-limb strength (13). Consistent with this developmental pattern, a recent large-scale study of 9,669 non-athletic adolescents aged 13–15 years found significant sex-by-age interactions across several physical fitness measures, including lower-body power and sprint performance, with larger age-related differences observed in boys than in girls (14). These findings highlight the importance of considering both upper- and lower-body manifestations of muscular fitness, as well as sex, when examining their associations with COD performance during the transition from childhood to adolescence.

Alongside muscular strength, flexibility represents another component of physical fitness that may be associated with COD performance. Adequate joint range of motion may facilitate the adoption of body positions required during acceleration, braking, and directional changes; however, the relationship between flexibility and COD performance remains less well established than that observed for muscular strength (15). Available evidence suggests that flexibility and COD performance may respond differently to physical training, indicating that these capacities should not be considered interchangeable manifestations of physical fitness (16). Nevertheless, evidence specifically examining the association between flexibility and pre-planned COD performance in children from general school populations remains limited. This uncertainty supports the need to examine flexibility alongside muscular strength and other fitness components when investigating COD performance in primary schoolchildren. Despite growing interest in multidirectional performance in young people, important gaps remain in the available evidence. Research in youth has frequently focused on athletic populations, particularly male participants and soccer players, limiting the generalizability of findings to children from the general school population (8, 11). Although previous studies in primary schoolchildren have examined several dimensions of physical fitness, comparatively little is known about how different fitness components are independently associated with pre-planned COD performance when considered simultaneously. This is particularly relevant for muscular strength and flexibility, as the association between lower-body strength and COD performance is supported by biomechanical and experimental evidence, whereas the relationship between flexibility and COD performance remains less clearly established. Furthermore, because muscular fitness develops differently in boys and girls across the transition from childhood to adolescence, it remains unclear whether sex modifies the associations between individual physical fitness components and COD performance. Examining these factors simultaneously in a non-selected sample of primary schoolchildren may therefore extend previous research by clarifying the independent associations of different physical fitness components with COD performance and determining whether these associations differ by sex.

Therefore, the present study aimed to: (i) examine sex differences in muscular strength, muscular endurance, flexibility, and change-of-direction (COD) performance; (ii) determine the adjusted associations between muscular strength, muscular endurance, flexibility, and COD performance; and (iii) examine whether sex moderates the associations between these physical fitness components and COD performance.

## 2 Methods

### 2.1 Study design and participants

This cross-sectional observational study was conducted among primary schoolchildren in Andalusia, Spain. Participants were recruited from two primary schools using non-probability convenience sampling and were enrolled across four grade levels, from the third to the sixth grade of primary education. A total of 395 children were initially contacted for participation. Of these, seven did not meet the eligibility criteria and two did not complete the physical fitness assessment protocol, resulting in a final analytical sample of 386 participants and an overall response rate of 97.7%.

The final sample comprised 176 boys (45.6%) and 210 girls (54.4%), aged 10 to 12 years, with a mean age of 11.15 ± 0.66 years. Participation was voluntary, and written informed consent was obtained from parents or legal guardians, together with assent from the children, prior to participation.

Children were eligible to participate if they were enrolled in primary education, were within the predefined age range, regularly participated in school physical education classes, and were able to safely complete the physical fitness assessments. Exclusion criteria included any medical, neurological, or musculoskeletal condition that could limit participation in physical activity or interfere with performance during the assessments. Children with temporary injuries or health conditions at the time of testing were also excluded. No missing data were present for the variables included in the analyses; therefore, all analyses were conducted using the complete dataset of 386 participants.

An *a priori* sample-size calculation was performed using G*Power version 3.1 for a two-tailed bivariate correlation analysis. Assuming a significance level of *α* = 0.05, a statistical power of 80%, and a small expected effect size (*r* = 0.15), a minimum sample of 340 participants was required. To account for an anticipated 10% loss due to incomplete questionnaires or invalid data, the target sample size was increased to 374 participants. The final analytical sample of 386 participants therefore exceeded the prespecified minimum sample size.

Because the original sample-size calculation was not specifically designed for the moderation analysis, an additional sensitivity analysis was conducted for the hierarchical regression model. With 386 participants, *α* = 0.05, 80% statistical power, four tested interaction terms, and 11 total predictors, the analysis was able to detect a minimum incremental effect size of approximately *f*^2^ = 0.031. Given the variance explained by the model before inclusion of the interaction terms (R^2^ = 0.626), this corresponds to an approximate detectable increase in explained variance of *Δ*R^2^ = 0.011. Thus, the available sample provided adequate power to detect small interaction effects, although very small moderation effects may have remained undetected.

### 2.2 Measures

#### 2.2.1 Sociodemographic and anthropometric characteristics

Sociodemographic information included age and sex. Age was recorded in years, and sex was categorized as boys or girls. Anthropometric measurements included body mass and height. Body mass was measured in kilograms using a calibrated digital scale, with participants wearing light clothing and no shoes. Height was measured in centimeters using a portable stadiometer, with participants standing barefoot in an upright position, with the head positioned in the Frankfort horizontal plane. Body mass index (BMI) was subsequently calculated as body mass in kilograms divided by height in meters squared (kg/m^2^).

#### 2.2.2 Handgrip strength

Handgrip strength was assessed using a digital hand dynamometer [TKK 5401 Grip-D (Takei Scientific Instruments, Tokyo, Japan)] and expressed in kilograms (kg). Prior to testing, the dynamometer was adjusted according to each participant's hand size to ensure a comfortable and standardized grip. Participants performed the test in a standing position, with the tested arm alongside the body, the elbow fully extended, and the wrist maintained in a neutral position. They were instructed to squeeze the dynamometer as strongly as possible for approximately 3–5 s, while avoiding compensatory movements and without allowing the dynamometer or the tested arm to contact the body.

Two maximal attempts were performed with each hand, alternating between the right and left sides, with an appropriate rest interval between attempts to minimize fatigue. The highest value obtained for each hand was retained. For the statistical analyses, the mean of the best values obtained for the right and left hands was calculated and used as the indicator of handgrip strength. Higher values indicated greater handgrip strength.

#### 2.2.3 Lower-body explosive strength

Lower-body explosive strength was assessed using the standing long jump test according to the procedures described in the ALPHA-Fitness test battery for children and adolescents (17). Participants stood behind a marked starting line with their feet approximately shoulder-width apart. From a standing position, they were instructed to jump forward as far as possible using a simultaneous two-foot take-off and landing. Arm swing and countermovement of the lower limbs were permitted to facilitate maximal performance. Participants were required to maintain balance upon landing without placing their hands on the floor or taking an additional step. Jump distance was measured in centimeters from the take-off line to the rearmost point of contact with the floor upon landing. Two maximal attempts were performed, with an appropriate recovery period between trials, and the best valid distance was retained for the statistical analyses. Greater distances indicated greater lower-body explosive strength.

#### 2.2.4 Muscular endurance

Muscular endurance was assessed using the 30-s sit-up test according to the procedures described in the EUROFIT test battery (18). Participants started in a supine position with their knees flexed and feet supported on the floor. They were instructed to perform as many correctly executed sit-ups as possible within 30 s, maintaining the standardized technique throughout the test. Each repetition required the participant to raise the upper body from the starting position and subsequently return to the initial position before beginning the next repetition. Only correctly completed repetitions were counted. The total number of valid repetitions performed within 30 s was recorded and used for the statistical analyses, with higher scores indicating greater muscular endurance.

#### 2.2.5 Flexibility

Flexibility was assessed using the sit-and-reach test according to the procedures described in the EUROFIT test battery (18). The test was performed using a 33-cm sit-and-reach box equipped with a sliding ruler on its upper surface. Participants sat on the floor with their knees fully extended, their feet approximately shoulder-width apart, and the soles of their feet placed against the front of the box. From this position, they were instructed to flex the trunk and reach forward with both arms as far as possible while keeping the knees extended. The maximal reach position was maintained for at least 2 s before the distance was recorded in centimeters. Two attempts were performed, and the best result was retained for the statistical analyses. Higher scores indicated greater flexibility.

#### 2.2.6 Change-of-direction performance

COD performance was assessed using the 4 × 10-m shuttle-run test included in the ALPHA-Fitness test battery (17). Two parallel lines were marked on the floor 10 m apart, and three sponges were used to complete the test. Participants started behind one of the lines with one sponge placed at the opposite line and two sponges positioned at the starting line. At the start signal, participants ran as quickly as possible to the opposite line, picked up the sponge, returned to the starting line, exchanged it for another sponge, and ran back to the opposite line. They then exchanged the sponge again and completed the final 10-m shuttle, resulting in a total distance of 40 m. Participants were instructed to perform the test at maximal speed throughout. Completion time was recorded in seconds, with lower values indicating better COD performance.

### 2.3 Procedure

All assessments were conducted during school hours by two trained researchers following standardized procedures. Before testing began, the researchers provided standardized verbal instructions for each assessment and demonstrated the correct execution of all tests to ensure that participants understood the procedures. Children were allowed to ask questions before performing each assessment.

Anthropometric and physical fitness assessments were completed during the same testing session. Before the physical fitness assessments, participants completed a standardized warm-up consisting of light running and dynamic joint-mobility exercises. A 15-min recovery period was provided between physical fitness tests to minimize the potential influence of fatigue on subsequent performance. Participants wore comfortable sports clothing and appropriate footwear throughout the assessments.

For the 4 × 10-m shuttle-run test, two parallel lines were marked 10 m apart. Completion time was measured manually using a handheld stopwatch. Each participant completed two trials, with a 5-min recovery interval between attempts, and the fastest completion time was retained for analysis. The test was performed on a clean, non-slip surface, and participants were instructed to complete each trial as quickly as possible.

### 2.4 Statistical analysis

Statistical analyses were performed using IBM SPSS Statistics for Windows, version 22.0 (IBM Corp., Armonk, NY, USA). Continuous variables were summarized using means and standard deviations, whereas categorical variables were expressed as frequencies and percentages. The distribution of continuous variables was examined prior to inferential analyses. Differences between boys and girls in age, body mass index (BMI), physical fitness components, and COD performance were examined using independent-samples Student's *t*-tests. Homogeneity of variance was assessed using Levene's test. Effect sizes for between-sex comparisons were estimated using Cohen's *d*. Raw BMI was used as a continuous covariate for statistical adjustment rather than for classification of weight status; age and sex were additionally included as separate covariates in the regression models. Pearson's correlation coefficients were calculated to examine bivariate associations between COD performance, handgrip strength, lower-body explosive strength, muscular endurance, flexibility, age, and BMI.

Hierarchical multiple linear regression analysis was subsequently performed to examine factors associated with COD performance. COD completion time was entered as the dependent variable. In Model 1, age, sex, and BMI were entered as covariates. In Model 2, handgrip strength, lower-body explosive strength, muscular endurance, and flexibility were additionally entered to examine their adjusted associations with COD performance beyond the covariates. Changes in the coefficient of determination (*Δ*R^2^) were used to quantify the additional variance explained by the physical fitness components. Unstandardized regression coefficients (B), standardized coefficients (*β*), 95% confidence intervals (CI), and *p* values were reported. To examine whether sex moderated the associations between physical fitness components and COD performance, continuous physical fitness variables were mean-centered and interaction terms between sex and each physical fitness component were calculated. These interaction terms were entered simultaneously in a third regression block. The change in explained variance (*Δ*R^2^) and the individual interaction coefficients were examined to determine the presence of moderation by sex. Regression assumptions were evaluated through residual diagnostics. Normality of residuals was assessed using a histogram and normal probability (P–P) plot, whereas linearity and homoscedasticity were evaluated by visual inspection of standardized residuals plotted against standardized predicted values. Multicollinearity was assessed using tolerance and variance inflation factor (VIF) values. Potentially influential observations were examined using studentized deleted residuals, Cook's distance, and leverage values. Statistical significance was set at *p* < 0.05 (two-tailed).

## 3 Results

### 3.1 Participant characteristics and sex-based comparisons

A total of 386 schoolchildren were included in the analysis, comprising 176 boys (45.6%) and 210 girls (54.4%). Descriptive characteristics and between-sex comparisons are presented in Table 1. No statistically significant differences between boys and girls were detected for age, height, body mass, BMI, any of the physical fitness measures, or COD performance (all *p* > 0.05). The estimated between-sex effect sizes were small, with all 95% confidence intervals including zero.

**Table 1:** Participant characteristics and physical fitness measures according to sex.

Variable	Boys (*n* = 176)	Girls (*n* = 210)	Mean difference (95% CI)	p	Cohen's d
Age (years)	11.20 ± 0.64	11.10 ± 0.67	0.105 (−0.028 to 0.237)	0.122	0.16
Height (cm)	151.06 ± 5.77	151.12 ± 5.68	−0.06 (−1.21 to 1.09)	0.915	−0.01
Body mass (kg)	45.03 ± 5.03	45.05 ± 4.90	−0.02 (−1.02 to 0.97)	0.962	0.00
BMI (kg/m^2^)	19.65 ± 0.73	19.65 ± 0.69	0.002 (−0.141 to 0.144)	0.984	0.00
Handgrip strength (kg)	18.59 ± 4.61	18.83 ± 4.79	−0.23 (−1.18 to 0.71)	0.630	−0.05
Lower-body explosive strength (cm)	123.24 ± 20.72	124.26 ± 22.42	−1.02 (−5.37 to 3.33)	0.646	−0.05
Trunk muscular endurance (repetitions)	19.94 ± 4.13	20.15 ± 4.43	−0.21 (−1.08 to 0.65)	0.625	−0.05
Flexibility (cm)	22.09 ± 5.81	21.91 ± 5.54	0.18 (−0.96 to 1.32)	0.759	0.03
Change-of-direction performance (s)	14.03 ± 0.71	13.93 ± 0.74	0.10 (−0.05 to 0.24)	0.190	0.13

Data are presented as mean ± standard deviation (SD). BMI, body mass index. Between-sex differences were assessed using independent-samples Student's t-tests. Mean differences were calculated as boys minus girls. Cohen's d was calculated as a measure of effect size.

### 3.2 Associations between physical fitness and COD performance

Pearson correlation analysis revealed significant associations between change–of–direction (COD) performance and all physical fitness variables assessed (Table 2). Lower–body explosive strength showed the strongest correlation with COD performance (r = −0.775, *p* < 0.001), followed by handgrip strength (r = −0.706, *p* < 0.001), flexibility (r = −0.534, *p* < 0.001), and trunk muscular endurance (r = −0.532, *p* < 0.001). Additionally, significant positive correlations were observed among the physical fitness variables (r = 0.430 –0.823, all *p* < 0.001). Neither age (r = 0.002, *p* > 0.05) nor BMI (r = −0.088, *p* > 0.05) showed a statistically significant correlation with COD performance.

**Table 2:** Pearson correlations between physical fitness characteristics and change-of-direction.

Variable	1	2	3	4	5	6	7
1. Lower-body explosive strength (cm)	—						
2. Trunk muscular endurance (repetitions)	.647***	—					
3. Flexibility (cm)	.571***	.430***	—				
4. Change-of-direction performance (s)	−.775***	−.532***	−.534***	—			
5. Handgrip strength (kg)	.823***	.674***	.567***	−.706***	—		
6. Age	.039	−.034	.000	.002	.033	—	
7. BMI	.075	.079	.061	−.088	.105*	.052	—

*N* = 386. Values are Pearson correlation coefficients (r). *p < .05; ***p < .001. BMI, body mass index.

### 3.3 Factors independently associated with COD performance

In Model 1, age, sex, and BMI explained 1.2% of the variance in COD performance, and the model was not statistically significant (*R*^2^ = 0.012, *p* = 0.194; Table 3). Adding the physical fitness components in Model 2 substantially increased the explained variance (*ΔR*^2^ = 0.614, *p* < 0.001), with the final model explaining 62.6% of the variance.

**Table 3:** performance hierarchical multiple linear regression analysis of factors associated with change-of-direction performance.

Model/Variable	B	95% CI for B	*β*	p	VIF
Model 1					
Age	0.001	−0.110 to 0.112	0.001	0.986	1.009
Sex	−0.098	−0.244 to 0.049	−0.067	0.191	1.006
Body mass index	−0.091	−0.193 to 0.012	−0.088	0.084	1.003
*Model 1 statistics*	*R^2^* *=* *0.012; Adjusted R^2^* *=* *0.004; F(3, 382)* *=* *1.577; p* *=* *0.194*				
Model 2					
Age	0.030	−0.039 to 0.098	0.027	0.397	1.018
Sex	−0.071	−0.162 to 0.019	−0.049	0.123	1.009
Body mass index	−0.022	−0.086 to 0.042	−0.022	0.493	1.015
Handgrip strength	−0.028	−0.046 to −0.010	−0.180	0.003	3.608
Lower-body explosive strength	−0.019	−0.023 to −0.015	−0.566	<0.001	3.418
Trunk muscular endurance	0.001	−0.013 to 0.016	0.007	0.878	1.942
Flexibility	−0.014	−0.024 to −0.004	−0.111	0.005	1.556
*Model 2 statistics*	*R^2^* *=* *0.626; Adjusted R^2^* *=* *0.619; ΔR^2^* *=* *0.614; ΔF(4, 378)* *=* *155.173, p* *<* *0.001; F(7, 378)* *=* *90.438, p* *<* *0.001*				

Dependent variable: change-of-directionperformance (4 × 10-m shuttle-run time, s); lower values indicate better performance. Model 1 included age, sex, and body mass index. Model 2 additionally included handgrip strength, lower-body explosive strength, muscular endurance, and flexibility. Sex was coded as 0 = boys and 1 = girls. B, unstandardized regression coefficient; β, standardized regression coefficient; CI, confidence interval; VIF, variance inflation factor; *Δ*R^2^, change in R^2^.

Lower-body explosive strength showed the strongest adjusted association with COD performance (*β* = −0.566, *p* < 0.001), followed by handgrip strength (*β* = −0.180, *p* = 0.003) and flexibility (*β* = −0.111, *p* = 0.005). Trunk muscular endurance was not significantly associated with COD performance after adjustment for the remaining variables. Age, sex, and BMI were also not statistically significant in the adjusted model. Given the strong correlation between handgrip strength and lower-body explosive strength (r = 0.823), their regression coefficients should be interpreted as adjusted associations reflecting the unique statistical contribution of each variable while holding the other predictors constant.

### 3.4 Moderating role of sex

No statistically significant interaction between sex and any of the physical fitness components was detected (Table 4). The inclusion of the four interaction terms explained an additional 0.3% of the variance in COD performance and did not significantly improve the model (*ΔR*^2^ = 0.003, *ΔF*(4, 374) = 0.643, *p* = 0.632). Regression diagnostic analyses indicated that the residual distribution was approximately normal, and visual inspection of standardized residuals against standardized predicted values did not indicate substantial departures from linearity or homoscedasticity. No highly influential observations were identified (maximum Cook's distance = 0.034; maximum centered leverage = 0.078; maximum studentized deleted residual = 2.876). Collinearity diagnostics indicated elevated VIF values for handgrip strength, lower-body explosive strength, and their corresponding interaction terms (VIFs approximately 8.4–8.7), consistent with the strong correlation between these fitness measures. Accordingly, the corresponding regression coefficients were interpreted cautiously as adjusted associations within the multivariable model.

**Table 4:** Moderating effect of sex on the associations between physical fitness components and change-of-direction performance.

Variable	B	95% CI for B	β	*p*	Tolerance	VIF
Age	0.025	−0.044 to 0.094	0.023	.479	.973	1.028
Sex	−0.072	−0.163 to 0.019	−0.049	.121	.991	1.009
BMI	−0.021	−0.087 to 0.044	−0.021	.519	.953	1.049
Handgrip strength	−0.031	−0.060 to −0.003	−0.201	.031	.115	8.716
Lower-body explosive strength	−0.016	−0.022 to −0.010	−0.478	<.001	.116	8.651
Trunk muscular endurance	−0.008	−0.030 to 0.014	−0.048	.471	.226	4.422
Flexibility	−0.017	−0.031 to −0.003	−0.132	.015	.339	2.947
Sex × Handgrip	0.004	−0.033 to 0.041	0.019	.837	.119	8.393
Sex × Lower-body explosive strength	−0.005	−0.013 to 0.003	−0.116	.205	.119	8.400
Sex × Trunk muscular endurance	0.017	−0.013 to 0.046	0.075	.266	.219	4.557
Sex × Flexibility	0.006	−0.014 to 0.026	0.035	.537	.303	3.299

B = unstandardized regression coefficient; CI = confidence interval; β = standardized regression coefficient. Continuous physical fitness variables were mean-centered before creating the interaction terms. Sex was coded as 0 = boys and 1 = girls. The dependent variable was change-of-direction performance measured as 4 × 10-m shuttle-run completion time; therefore, lower values indicate better performance.

## 4 Discussion

The present study examined the associations between multiple components of physical fitness and COD performance in primary schoolchildren, as well as potential between-sex differences and the moderating role of sex. Overall, lower-body explosive strength showed the strongest adjusted association with COD performance, while handgrip strength and flexibility also remained associated with performance in the multivariable model. No statistically significant between-sex differences or sex-related moderation effects were detected. These findings highlight the multidimensional nature of COD performance and the particularly strong association observed with lower-body explosive strength.

### 4.1 Sex differences in physical fitness and COD performance

No statistically significant differences between boys and girls were detected in any of the physical fitness components or in COD performance. This result is particularly relevant given that sex-related differences in physical fitness have been reported during childhood and adolescence, although their magnitude and emergence appear to vary according to age, biological maturation, physical activity exposure, and the specific fitness component examined.

Previous studies in school-aged populations have not provided completely uniform findings. Teich et al. (10), for example, reported sex-related differences in several components of physical fitness among primary schoolchildren, including measures related to speed, coordination, and lower-limb power. Importantly, developmental patterns varied according to sex and school grade, particularly around the transition from late childhood to early adolescence. Similarly, Aimar et al. (19), in a large sample of Argentine schoolchildren aged 9–12 years, reported better overall physical fitness performance in boys, including standing long jump and 4 × 10-m shuttle-run performance. These findings differ from the present results, in which no statistically significant between-sex differences were detected, and highlight that sex-related differences in physical fitness may vary across school-aged populations (20).

One possible explanation may lie in the developmental characteristics of the present sample. At approximately 11 years of age, considerable interindividual variability in biological maturation may exist, and chronological age does not necessarily provide a complete representation of maturational status. Longitudinal evidence indicates that the developmental trajectories of handgrip strength, lower-body power, and shuttle-run performance vary in relation to biological maturation and sex (21). Moreover, recent meta-analytic evidence indicates that sex differences in muscular strength are already detectable during childhood but become substantially more pronounced during adolescence, particularly following male pubertal development (13). Therefore, the lack of statistically significant between-sex differences in the present study may partly reflect the developmental stage of the sample, during which sex-related differences may still be emerging (22). This interpretation should, however, be considered cautiously because biological maturation was not directly assessed in the present study. Given that both between-sex differences and the moderating role of sex were central aims of the study, the absence of a direct measure of maturation limits the extent to which the present findings can distinguish sex-related associations from differences related to maturational status.

### 4.2 Muscular strength and COD performance

Lower-body explosive strength showed the strongest association with COD performance, both at the bivariate level and in the multivariable model. This finding is biomechanically plausible, as rapid changes of direction require deceleration, absorption of braking forces, and subsequent force generation to reaccelerate the center of mass in a new direction (7, 9). The observed association is also consistent with the shared neuromuscular requirements of jumping, acceleration, and change-of-direction tasks. Previous evidence in primary schoolchildren has reported associations among lower-limb power, sprinting, and coordination-related performance (10), supporting substantial overlap among these motor fitness components during childhood. In the present study, lower-body explosive strength remained strongly associated with COD performance after adjustment for handgrip strength, muscular endurance, flexibility, age, sex, and BMI.

Evidence from intervention studies further supports a close relationship between lower-limb explosive capacity and change-of-direction performance, although most of this research has been conducted in young athletes rather than general school populations. Oliver et al. (11), in a systematic review and meta-analysis of highly trained male youth soccer players, found that strength, plyometric, and combined training produced significant improvements in lower-body strength, horizontal power, acceleration, and change-of-direction speed. More recently, Zheng et al. (23) reported that plyometric training significantly improved both jumping performance and change-of-direction performance in adolescent soccer players, with moderate effects for both outcomes. Similarly, a recent network meta-analysis involving adolescent team-sport athletes showed that strength-oriented interventions, particularly eccentric and combined training approaches, can improve change-of-direction performance (24). Although these intervention studies involved older and more sport-specific populations than the present sample, they provide complementary evidence regarding the relationship between lower-body strength and power and performance in tasks requiring rapid changes of direction and should not be directly extrapolated to the present cross-sectional sample of primary schoolchildren.

Handgrip strength was also strongly correlated with COD performance and remained associated with performance after adjustment for the other physical fitness components, although its standardized association was considerably smaller than that of lower-body explosive strength (25). Given that handgrip strength is not directly involved in propulsion or braking during the 4 × 10-m shuttle run, this association is unlikely to represent a direct biomechanical effect. Rather, handgrip strength may function as a broader indicator of muscular fitness and neuromuscular development. Importantly, handgrip strength and lower-body explosive strength were strongly correlated (r = 0.823), indicating substantial shared variance between these measures. Therefore, their regression coefficients should be interpreted as adjusted associations conditional on the other predictors in the model rather than as fully distinct or independent contributions. This overlap may also partly explain the attenuation of the association for handgrip strength in the multivariable model. Nevertheless, lower-body explosive strength retained the larger standardized association with COD performance, consistent with the specific mechanical demands of acceleration, deceleration, and reacceleration during the 4 × 10-m shuttle run. Muscular endurance

Muscular endurance showed a moderate bivariate association with COD performance; however, this association was no longer statistically significant when the remaining physical fitness components were simultaneously considered. Trunk muscular endurance was also correlated with handgrip strength and lower-body explosive strength, suggesting that its bivariate association with COD performance may partly reflect variance shared with other dimensions of muscular fitness. Similar studies in schoolchildren have shown that muscular strength, endurance, flexibility, and motor performance represent related but distinct dimensions of physical fitness (26, 27). These findings highlight the importance of distinguishing bivariate from adjusted associations when correlated components of physical fitness are considered concurrently.

### 4.3 Flexibility and COD performance

Flexibility was associated with COD performance and remained statistically significant in the multivariable model, although its adjusted association was considerably smaller than that of lower-body explosive strength. This finding is noteworthy because the relationship between flexibility and change-of-direction performance in young populations remains less clearly established than that of muscular strength and power.

Previous evidence regarding this relationship is not entirely consistent. In a recent study of elite male youth soccer players aged 12–14 years, Palco et al. (28) simultaneously assessed flexibility using the sit-and-reach test, lower-body explosive strength using the standing broad jump, and change-of-direction performance using the T-Test. Although all three capacities improved significantly across the competitive season, sit-and-reach performance was not significantly associated with change-of-direction time at the end of the season (*r* = −0.15, *p* = 0.31), whereas standing broad jump performance showed a significant association with change-of-direction performance (*r* = −0.41, *p* = 0.005). Moreover, changes in these capacities across the season were largely independent of one another. These findings contrast with the present results, in which flexibility remained associated with COD performance after adjustment for the other physical fitness components, and suggest that the relationship between flexibility and change-of-direction performance may vary according to factors such as age, population characteristics, task demands, or the specific measure of COD performance employed.

From a biomechanical perspective, joint range of motion may be relevant to the positions adopted during acceleration, deceleration, and directional transitions. However, flexibility should not be considered in isolation when examining COD performance, as change-of-direction ability involves multiple neuromuscular and biomechanical factors (7). Accordingly, the weaker standardized association of flexibility compared with lower-body explosive strength in the present study is consistent with the substantial force- and power-related demands of the 4 × 10-m shuttle run.

Importantly, the present findings should be interpreted in light of the measure used to assess flexibility. The sit-and-reach test primarily reflects hamstring extensibility and posterior-chain flexibility and does not capture all aspects of the multiplanar, joint-specific mobility required during rapid changes of direction (29). Therefore, the observed association should not be interpreted as evidence that increasing sit-and-reach flexibility would necessarily improve COD performance. Rather, sit-and-reach performance should be interpreted as a specific flexibility measure within the broader physical fitness profile examined in this study. Further longitudinal and intervention studies using joint-specific measures of mobility are needed to clarify the nature and direction of the association between flexibility and COD performance in school-aged children (30).

### 4.4 Strengths and limitations

Several strengths should be considered. The study included a relatively large sample of primary schoolchildren of both sexes and assessed multiple components of physical fitness using standardized field-based procedures. In addition, the hierarchical analytical approach allowed the adjusted association of each physical fitness component with COD performance to be examined after controlling for age, sex, and BMI. The inclusion of interaction terms further allowed the potential moderating role of sex to be formally tested rather than inferred from separate subgroup analyses.

However, the findings should also be interpreted in light of several limitations. First, the cross-sectional design precludes establishing causal relationships. Although muscular strength and flexibility were associated with COD performance, it cannot be concluded that improving these capacities would necessarily result in corresponding improvements in COD performance. Second, biological maturation, habitual physical activity, and participation in organized sport were not assessed. These factors may be associated with both physical fitness and COD performance and therefore represent potential sources of residual confounding. This limitation is particularly relevant given the age range examined, during which children of the same chronological age may differ substantially in maturational status and exposure to physical activity. Future studies should incorporate measures of biological maturation and habitual physical activity and characterize participation in organized sports to better account for their potential influence on these associations.

Third, COD performance was assessed using the 4 × 10-m shuttle run, which is a standardized and practical field test of pre-planned change-of-direction ability. However, this task does not incorporate a reactive or decision-making component in response to an external stimulus and therefore should not be interpreted as a measure of reactive agility. Fourth, handgrip strength should be interpreted primarily as an indicator of general muscular fitness rather than as a direct measure of the muscular forces involved in change-of-direction performance. Finally, information identifying participants’ schools or individual classrooms was not retained in the analytical dataset. Therefore, potential clustering of participants within schools or classrooms could not be explicitly accounted for in the statistical analyses. Future studies should retain school and classroom identifiers and consider multilevel or cluster-adjusted analytical approaches where appropriate.

## 5 Conclusion

The present study showed that physical fitness components, particularly lower-body explosive strength, were strongly associated with COD performance in primary schoolchildren. Lower-body explosive strength showed the strongest adjusted association with 4 × 10-m shuttle-run performance, while handgrip strength and flexibility showed smaller but statistically significant adjusted associations. In contrast, trunk muscular endurance was associated with COD performance at the bivariate level but was no longer statistically significant when the other physical fitness components were considered simultaneously.

No statistically significant between-sex differences in physical fitness or COD performance were detected, and no statistically significant sex-related moderation effects were observed for the associations between physical fitness components and COD performance. These findings should be interpreted considering that biological maturation, habitual physical activity, and participation in organized sport were not assessed and may have influenced the observed associations. Overall, the results highlight the strong association between lower-body explosive strength and COD performance in primary schoolchildren, while supporting the multidimensional nature of change-of-direction performance. Longitudinal and intervention studies incorporating measures of biological maturation, habitual physical activity, and sport participation are needed to clarify the direction of these relationships and determine whether changes in specific physical fitness components are accompanied by changes in COD performance.

## Data Availability

The datasets generated and/or analyzed during the current study are not publicly available because they contain individual-level data from minors and are subject to privacy, ethical, and data-protection restrictions.
